# C-kit signaling promotes proliferation and invasion of colorectal mucinous adenocarcinoma in a murine model

**DOI:** 10.18632/oncotarget.4815

**Published:** 2015-09-02

**Authors:** Jun Tan, Shu Yang, Ping Shen, Haimei Sun, Jie Xiao, Yaxi Wang, Bo Wu, Fengqing Ji, Jihong Yan, Hong Xue, Deshan Zhou

**Affiliations:** ^1^ Department of Histology and Embryology, School of Basic Medical Sciences, Capital Medical University, Beijing 100069, P. R. China; ^2^ Beijing Key Laboratory of Cancer Invasion and Metastasis Research, Beijing 100069, P. R. China; ^3^ Cancer Institute of Capital Medical University, Beijing 100069, P. R. China

**Keywords:** colorectal mucinous adenocarcinoma, C-kit, ETV4, murine model

## Abstract

It was reported that the receptor tyrosine kinase (RTK) family often highly expressed in several mucinous carcinomas. In the present study, we established a murine model of colorectal mucinous adenocardinoma (CRMAC) by treating C57 mice [both wild type (WT) and loss-of-function *c-kit* mutant type (Wads^−/−^)] with AOM+DSS for 37 weeks and found that c-kit, a member of RTK family, clearly enhanced the tumor cell proliferation by decreasing p53 and increasing cyclin D1 through AKT pathway. Significantly, c-kit strongly promoted tumor cell invasiveness by increasing ETV4, which induced MMP7 expression and epithelial-mesenchymal transition (EMT) via ERK pathway. *In vitro* up- or down-regulating c-kit activation in human colorectal cancer HCT-116 cells further consolidated these results. In conclusion, our data suggested that the c-kit signaling obviously promoted proliferation and invasion of CRMAC. Therefore, targeting the c-kit signaling and its downstream molecules might provide the potential strategies for treatment of patients suffering from CRMAC in the future.

## INTRODUCTION

Colorectal cancer is the third most common cancer and the fourth leading cause of cancer death, accounting for approximately 1.36 million new cases and 700 thousand deaths each year [1]. Colorectal mucinous adenocarcinoma (CRMAC) is a subtype of colorectal adenocarcinoma, accounting for 15–20% of all colorectal cancer patients. CRMAC is characterized by abundant mount of extracellular mucin containing malignant tumor cells [2–4]. Compared with common colorectal adenocarcinoma, patients suffering from CRMAC often show high risk of metastasis and poor 5-years survival rate after treated with traditional chemotherapy and surgical resection [3, 4]. The main reason underlying the poor prognosis is the undefined mechanism of CRMAC invasiveness.

Recently, receptor tyrosine kinase (RTK) family has been attracting more attention because it may promote the development of multiple mucinous carcinomas. For example, human epidermal growth factor receptor-2 (HER2) was overexpressed in mucinous epithelial ovarian cancer [5]. Inhibition of insulin-like growth factor-1 receptor (IGF-1R) could overcome the resistance of mucinous lung adenocarcinoma to gefitinb [6]. Using a novel CRMAC cell line (COLM-6), Yamachika et al. found that this cell line expressed epidermal growth factor receptor (EGFR) and HER2, and exogenous EGF, transforming growth factor-α (TGF-α) and heregulin significantly stimulated cell growth [7]. Increased expression of c-kit was detected in intraductal papillary mucinous pancreatic neoplasm [8]. C-kit, a member of the RTK family, can be activated by its ligand, stem cell factor (SCF). The activation of c-kit signaling plays an important role in the development of many kinds of tumors, such as hematopoietic cell tumor, small cell lung cancer, melanoma, gastrointestinal stromal tumor (GIST) and colorectal cancer [9–11]. It has been reported that colorectal cancer tissues overexpressing c-kit often showed a stronger capacity of proliferation, higher risk of metastasis and poorer prognosis than other subtypes of colorectal cancer [12]. These worse clinical outcomes were possibly attributed to c-kit/SCF autocrine and/or paracrine loop, which enhanced proliferation and invasion of colorectal cancer through its downstream signaling molecules [13–15].

The members of ETS transcription factor family, especially ETV1, ETV4 and ETV5, have also been suggested to be able to facilitate cancer invasion and metastasis by activating gene transcriptions which encoded matrix metalloproteases (MMPs) [16–19], and promoting epithelial-mesenchymal transition (EMT) [20]. A recent study further showed that ETV1 could be up-regulated by c-kit signaling and helpful for the growth and survival of GIST [11]. It's worth noting that ETV4, a homologue of ETV1, was elevated in colorectal cancer tissues and enhanced tumor invasion [21, 22].

In the light of previous studies, we hypothesized that c-kit signaling could increase ETV4 expression and promote tumor cell invasiveness in CRMAC. In the present study, by using a murine model of CRMAC, in which C57 wild type and loss-of-function *c-kit* mutant Wads^−/−^ type mice were treated with AOM+DSS for longer time than usual, we showed that the c-kit signaling evidently promoted tumor growth by decreasing p53 and increasing cyclin D1. Furthermore, c-kit signaling reinforced CRMAC invasion by increasing ETV4 which subsequently enhanced MMP-7 expression and induced EMT progression. Similar results were also obtained in human colorectal cancer HCT-116 cells by activating or inhibiting c-kit activation.

## RESULTS

### Establishment of CRMAC murine model

**Figure 1:**
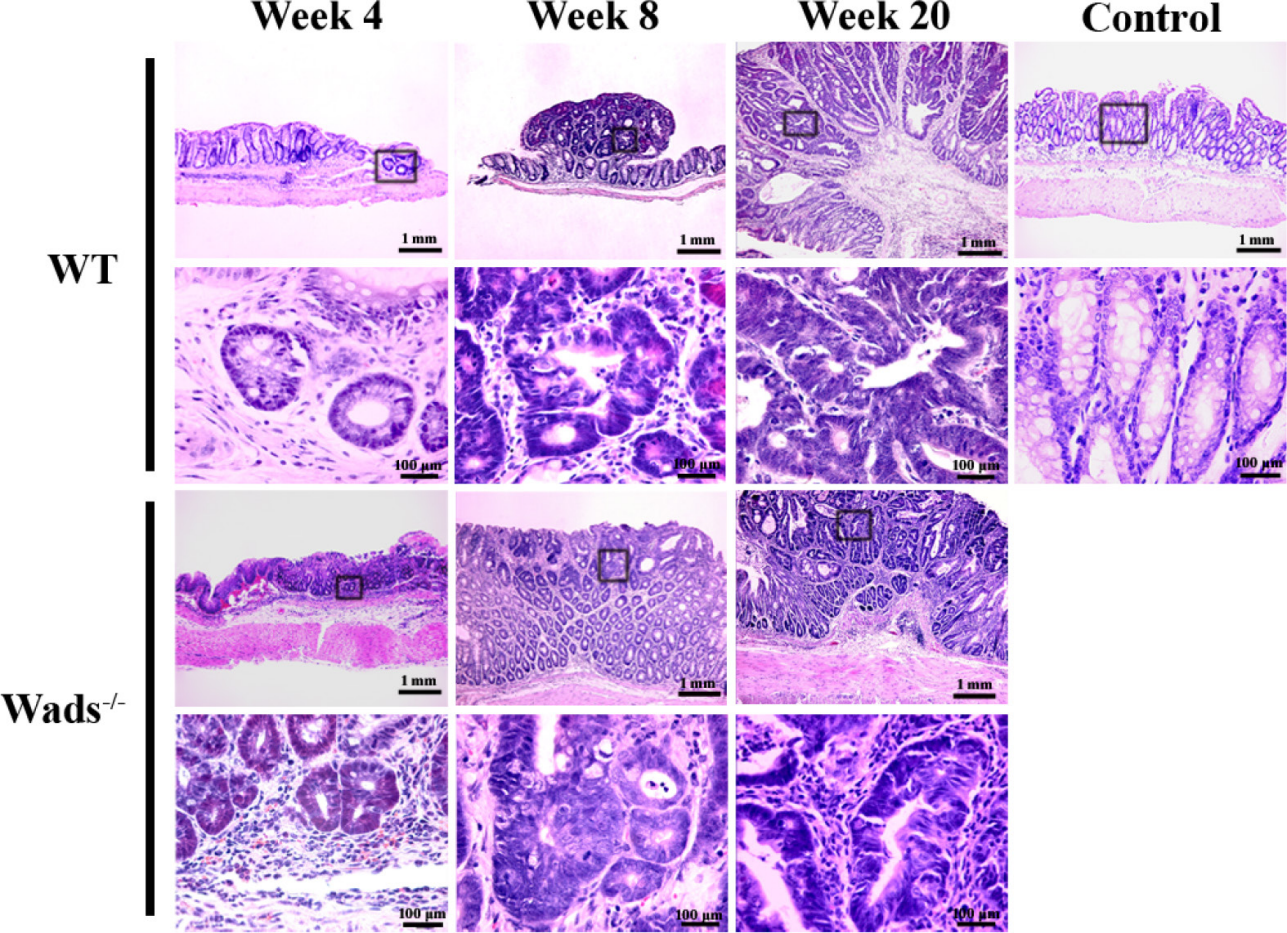
Establishment of CRMAC murine model in WT and Wads^−/−^ mice Mice were sacrificed 4, 8 and 20 weeks after AOM injection, respectively. Sections obtained from model mice and normal control mice were stained with HE for histopathological assessment. At week 4, both WT and Wads^−/−^ mice showed dysplasia of colonic glands. Adenomas were observed at week 8. At week 20, typical adenocarcinoma appeared both in WT mice and Wads^−/−^ mice. Views in the frames of upper panel are zoomed in in lower panel.

**Figure 2:**
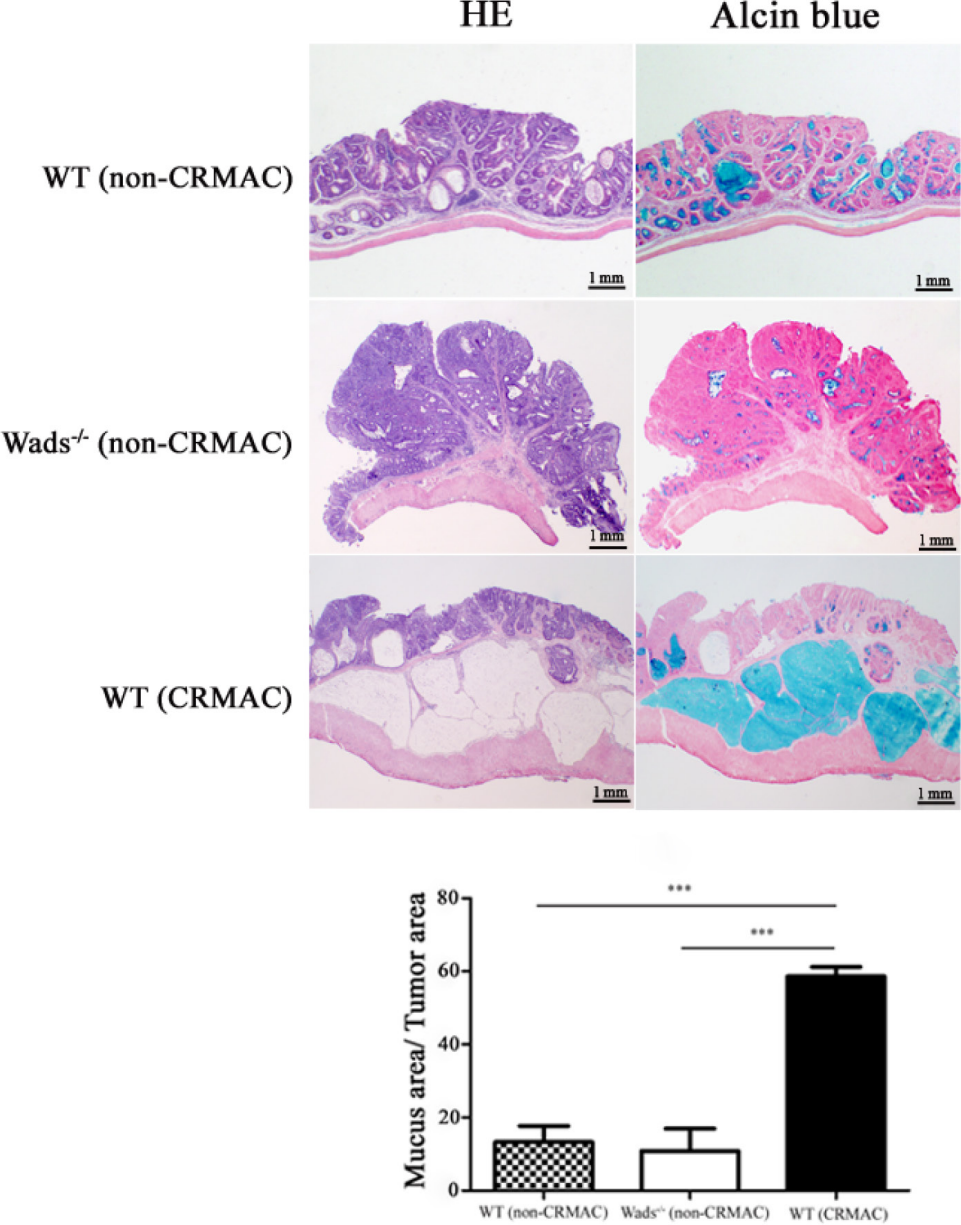
At week 37, adenocarcinomas developed CRMAC in half of WT mice, identified by HE and Alcian blue staining to assess the percentage of the mucus area (≥50% of the total tumor area) The rest WT and all Wads^−/−^ mice displayed no or few mucus that were defined as non-CRMAC (***P < 0.001).

We used WT mice and Wads^−/−^ mice to establish colorectal cancer model by AOM+DSS treatment as previously described [23]. Four weeks after AOM injection, dysplastic glands or aberrant crypt focuses were found in distal colon (Figure 1). At week 8, adenomas were observed, in which atypical glands showed apparent “back to back” and disordered arrangement of gland cells (Figure 1). At week 20, typical adenocarcinoma appeared with plenty of poorly differentiated dysplastic glands and nuclear divisions (Figure 1). Notably, at week 37, above half of WT mice had colorectal adenocarcinomas with abundant mount of extracellular mucin indicated by HE and Alcian blue staining, which met the criteria of CRMAC diagnosis (≥50% of the total tumor area) (Figure 2). Whereas, Wads^−/−^ mice did not have CRMAC although there were less content of mucus in the adenocarcinomas (Figure 2 and Supplementary Table S1). The groups and numbers of mice were indicated in Table 1. Our data suggest that AOM+DSS treatment for 37 weeks is able to induce the occurrence of CRMAC in WT mice.

**Table 1:** Animal groups

Number Group	Week 4	Week 8	Week 20	Week 37
WT model	10	10	10	CRMAC: 8, Non-CRMAC: 7
Wads^−/−^ model	5	5	5	CRMAC: 0, Non-CRMAC: 5
Control	—	—	—	5

Adult WT (*n* = 45) and Wads^−/−^ (*n* = 20) mice (8–10 weeks old, 19–22 g) were intraperitonealy injected with AOM once and followed by three periods of 2.5% DSS in drink water. For the controls, WT mice (*n* = 5) received an injection of normal saline and had free access to distilled water. Mice were weighed twice weekly during the period of the experiments and sacrificed at the indicated time points (4 weeks, 8 weeks, 20 weeks and 37 weeks) after AOM injection.

### C-kit is overexpressed in CRMAC

**Figure 3:**
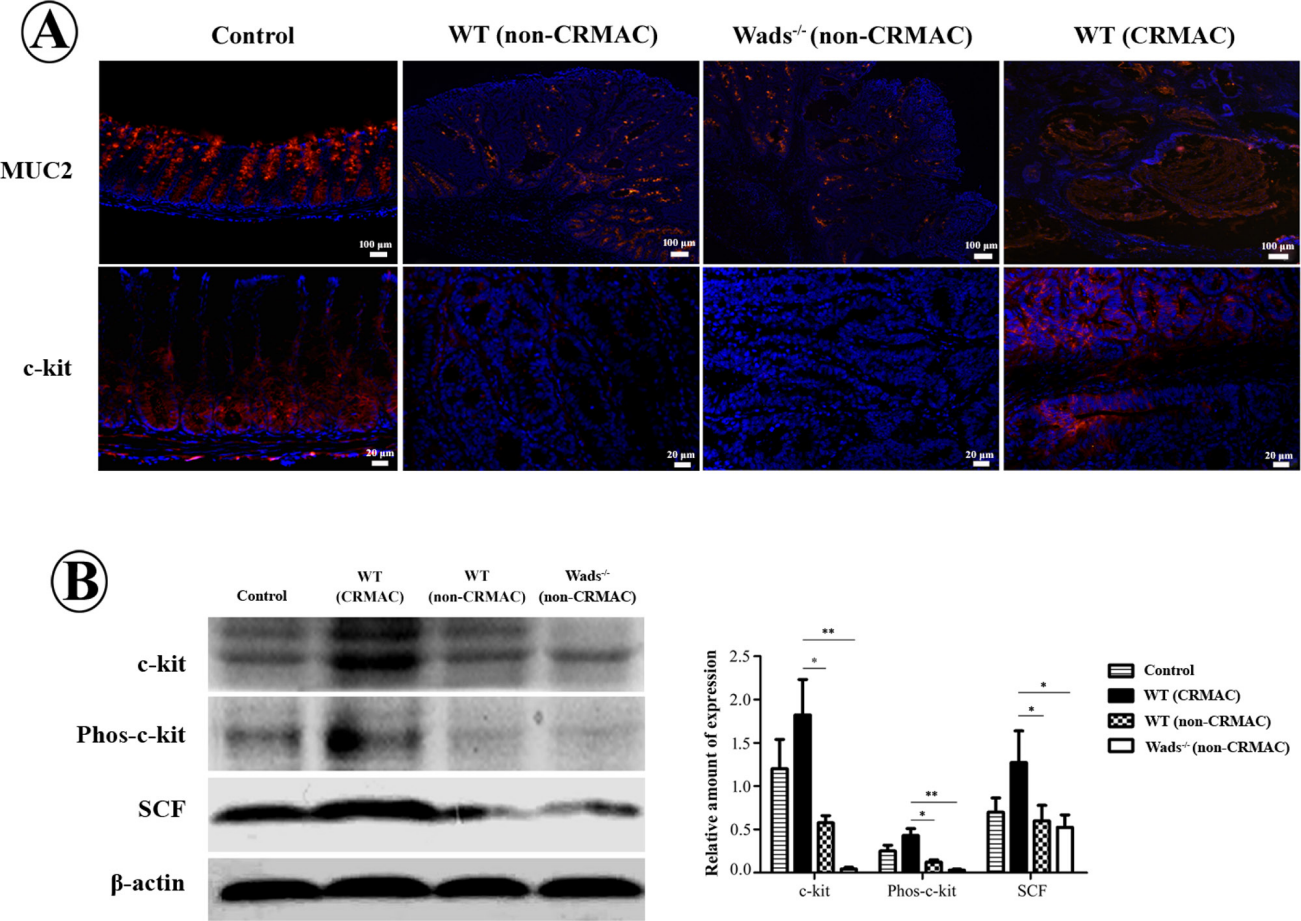
**A.** Immunofluorescence staining showed that in normal colonic mucosa MUC2 was expressed in goblet cells and c-kit in intestinal crypts. MUC2 was filled in mucus cavity in CRMAC. MUC2 and c-kit were increased in CRMAC of WT mice compared with those in non-CRMAC of WT mice and Wads^−/−^ mice at week 37. **B.** Western blotting showed that CRMAC in WT mice highly expressed c-kit, phosphorylated c-kit and its ligand SCF compared with non-CRMAC in WT mice and Wads^−/−^ mice did at week 37 (*P < 0.05, **P < 0.01).

It has been demonstrated that the mucus in tumor tissue is characterized by MUC2 and obviously increased in CRMAC [3]. Here, we used immunofluorescence technique to determine the phenotype of mucus in adenocarcinomas developed in the murine models. The results showed that most of mucus were MUC2 positive (Figure 3A), similar to that in CRMAC patients [3]. Remarkably, c-kit and its ligand SCF were significantly elevated in WT mice with CRMAC than those in WT and Wads^−/−^ mice with non-CRMAC (*P* < 0.05 or 0.01, Figure 3A and 3B). Furthermore, phosphorylated c-kit was increased in WT mice with CRMAC compared with WT and Wads^−/−^ mice with non-CRMAC, indicating that the c-kit signaling was highly activated (*P* < 0.05 or 0.01, Figure 3B). However, no difference in c-kit, phosphorylated c-kit, SCF and MUC2 was observed between WT and Wads^−/−^ mice with non-CRMAC (*P* < 0.05 Figure 3A and 3B). Thus, in the following experiments, we focused on the WT mice with CRMAC and the Wads^−/−^ mice with non-CRMAC.

### C-kit promotes proliferation of CRMAC

**Figure 4:**
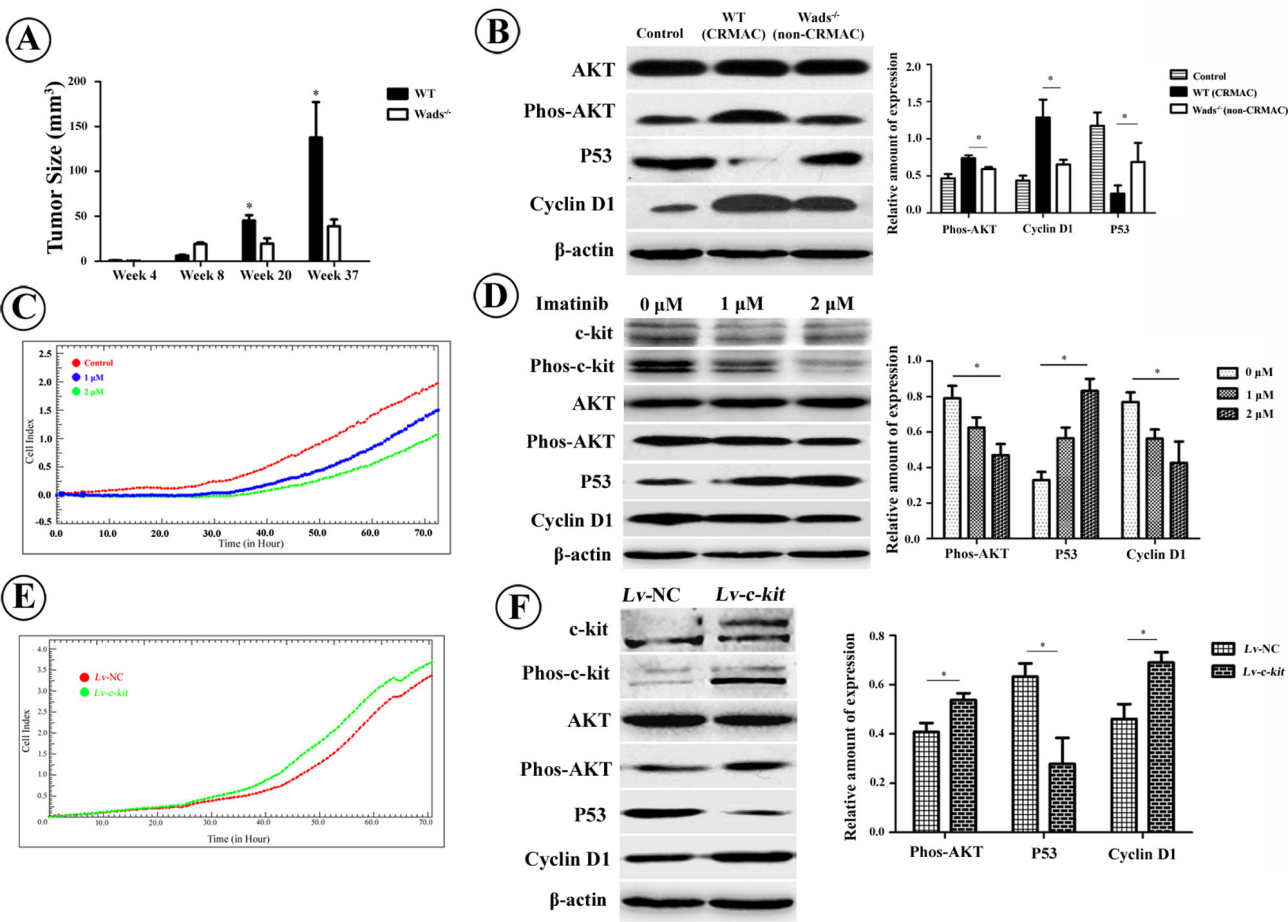
**A.** Compared with Wads^−/−^ mice, tumors grew much faster in WT mice starting from 20 weeks after AOM injection (*P < 0.05, **P < 0.01). **B.** At week 37, compared with non-CRMAC in Wads^−/−^ mice, AKT phosphorylation and cyclin D1 expression were highly increased whereas p53 expression was significantly decreased in CRMAC of WT mice (*P < 0.05). **C.** Imatinib treatment caused a dose-dependent proliferative suppression of HCT-116 cells recorded by RTCA. **D.** Imatinib treatment for 36 hrs attenuated activation of c-kit and AKT. Under the same condition, p53 was increased whereas cyclin D1 was decreased in a dose-dependent manner (*P < 0.05). **E.** Overexpression of c-kit in HCT-116 cells significantly promoted cellular proliferation in the presence of exogenous rhSCF (10 ng/ml). **F.** Hyperexpressed c-kit resulted in AKT activation which also reduced p53 and enhanced cyclin D1.

At week 20 and 37, the tumor size in WT mice were clearly larger than those in Wads^−/−^ mice (*P* < 0.05, Figure 4A), suggesting that c-kit played a role in promoting proliferation of tumor cells. Since PI3K/AKT pathway is involved in cell proliferation, we detected the AKT and its downstream p53 and cyclin D1. High level of phosphorylated AKT was detected in WT mice with CRMAC (*P* < 0.05, Figure 4B). We also found decreased p53 while increased cyclin D1 in WT mice with CRMAC compared with those in Wads^−/−^ mice with non-CRMAC at week 37 (*P* < 0.05, Figure 4B). *In vitro* treatment with imatinib to block c-kit activity resulted in proliferative suppression of HCT-116 cells (Figure 4C), simultaneously decreased AKT phosphorylation and cyclin D1 but increased p53 (*P* < 0.05, Figure 4D). In contrast, overexpression of c-kit by transfecting with Lv-*c-kit* construct significantly accelerated the proliferation of HCT-116 cells in the presence of exogenous rhSCF (Figure 4E), which might be owing to the increased phosphorylated AKT and cyclin D1, and decreased p53 (*P* < 0.05, Figure 4F). In line with previous studies [10, 13, 24], it is reasonable to consider that c-kit promotes proliferation of CRMAC through activating AKT pathway.

### C-kit promotes CRMAC invasion through increasing ETV4 expression

**Figure 5:**
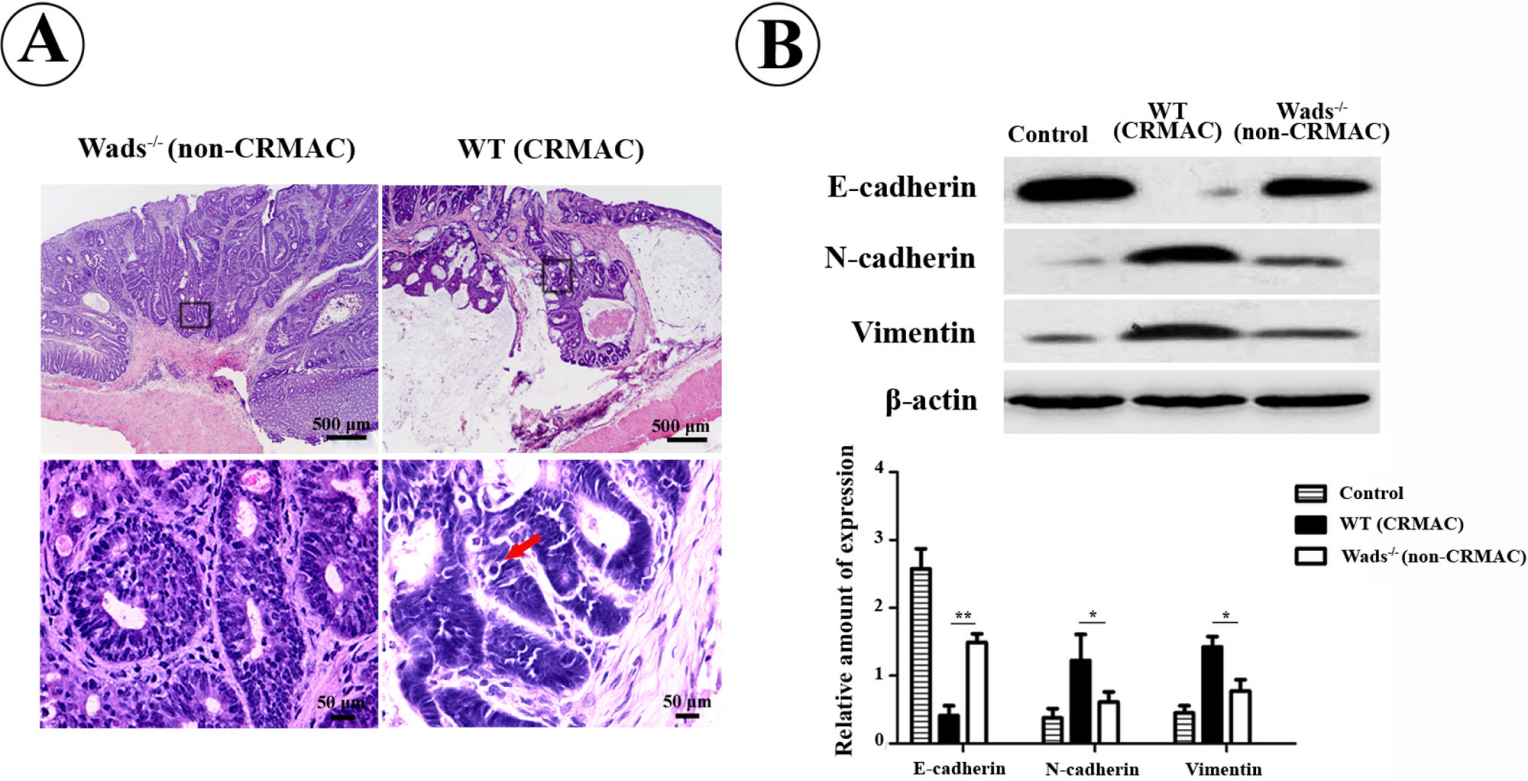
CRMAC in WT mice showed more invasiveness than non-CRMAC in Wads^−/−^ mice did at week 37 **A.** Tumor cells and mucinous extended beyond the basement membrane of mucosa and invaded into submucosa in WT mice, in which tumors developed more atypical glands and displayed more pathologic mitoses (red arrow). Views in the frames of upper panel are zoomed in in lower panel. **B.** EMT was strikingly elevated in CRMAC compared with non-CRMAC indicated by decreased E-cadherin and increased N-cadherin and vimentin. (*P < 0.05,**P < 0.01).

Previous studies have elucidated that CRMAC often exhibited high-grade malignancy with a strong invasive capacity [3, 4]. Accordantly, we found that CRMAC cells that highly expressed c-kit were more poorly differentiated, indicated by more atypical glands and pathologic mitoses compared with non-CRMAC at week 37 (Figure 5A). Remarkably, CRMAC cells extended beyond the basement membrane of intestinal mucosa and invaded into submucosa, even closed to the circular muscle layer (Figure 5A). On the contrary, the basement membrane was intact and no obvious tumor cell infiltration was observed in non-CRMAC (Figure 5A). In concomitance with the stronger invasiveness, CRMAC displayed a higher level of EMT predicated by decreased E-cadherin and increased N-cadherin and vimentin when compared with non-CRMAC (*P* < 0.05 or 0.01, Figure 5B). These findings suggested that c-kit signaling probably enhanced CRMAC invasiveness.

**Figure 6:**
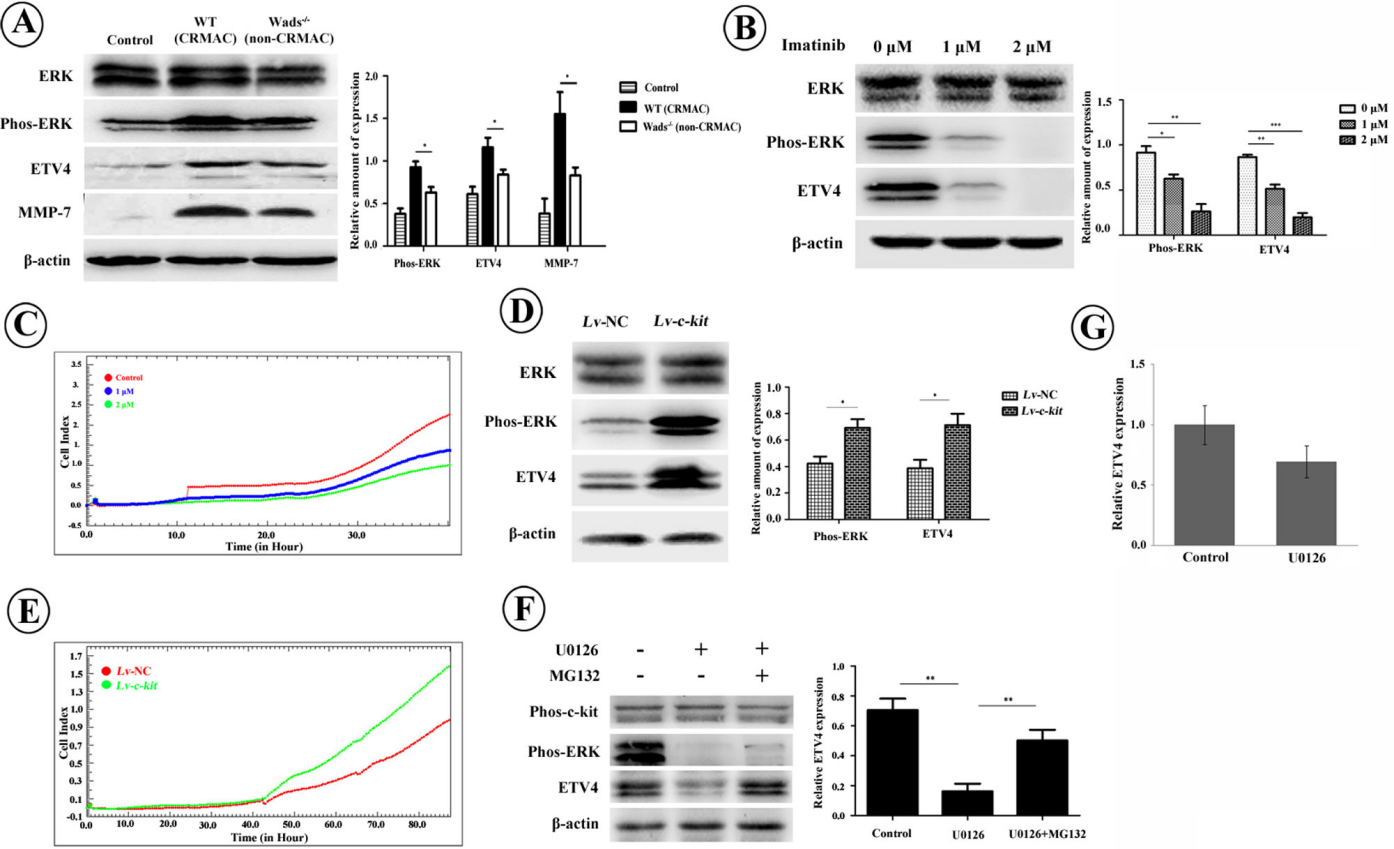
**A.** Compared with non-CRMAC in Wads^−/−^ mice and normal mucosa in controls, ETV4 and MMP-7 were elevated along with activated ERK pathway in CRMAC of WT mice at week 37 (*P < 0.05). **B.** Imatinib treatment for 36 hrs inactivated ERK and down-regulated ETV4 in HCT-116 cells in a dose-dependent manner (*P < 0.05, **P < 0.01, ***P < 0.001). **C.** Imatinib treatment repressed invasion of HCT-116 cells monitored by RTCA. **D.** Overexpression of c-kit increased phosphorylated ERK and ETV4 in HCT-116 cells in the presence of rhSCF (10 ng/ml) for 36 hrs (*P < 0.05). **E.** Overexpression of c-kit in the presence of rhSCF (10 ng/ml) enhanced invasive ability of HCT-116 cells recorded by RTCA. **F.** HCT-116 cells were treated with MEK inhibitor, U0126 (10 μM), for 2 hrs with or without proteosome inhibitor MG132 (20 μM). U0126 treatment significantly inhibited ERK activation and ETV4 expression whereas additional MG132 reversed the decrease of ETV4 (*P < 0.05, **P < 0.01). **G.** U0126 treatment did not significantly alter mRNA level of ETV4 (*P* > 0.05).

Recent studies have shown that ETV4 could enhance tumor cell invasiveness by increasing MMPs and inducing EMT [20–22]. Here, as shown in Figure 6A, ETV4 and one of its targets, MMP-7, were markedly increased in CRMAC which overexpressed c-kit (*P* < 0.05). Interestingly, highly phosphorylated ERK was also detected in CRMAC (*P* < 0.05), suggesting that ERK pathway was activated in the c-kit-induced invasiveness of CRMAC. We further carried out *in vitro* experiments in HCT-116 cells, which simultaneously express endogenous c-kit, SCF and ETV4 (Supplementary Figure S1). Again, blockage of c-kit activity by imatinib evidently inhibited ERK phosphorylation and ETV4 expression in HCT-116 cells (*P* < 0.05, 0.01, or 0.001, Figure 6B). Correspondingly, invasive capacity of HCT-116 cells was significantly suppressed (*P* < 0.05, Figure 6C). In contrast, overexpression of c-kit significantly increased ERK phosphorylation and ETV4 expression in HCT-116 cells (*P* < 0.05, Figure 6D). As expected, HCT-116 cells overexpressing c-kit showed a stronger invasiveness than those of controls (*P* < 0.05, Figure 6E). To confirm the role of ERK in the ETV4 expression, we treated HCT-116 cells with the MEK inhibitor, U0126. We found decreased ETV4 protein level (*P* < 0.01, Figure 6F) rather than mRNA expression (*P* > 0.05, Figure 6G), and the U0126-reduced ETV4 protein was rescued by MG132 treatment, an inhibitor of proteosomal degradation (*P* < 0.01, Figure 6F). These results implied that the phosphorylated ERK might protect ETV4 from proteosomal degradation instead of promoting ETV4 transcription. Collectively, these data suggest that c-kit could enhance tumor cell invasion by promoting ETV4 expression through activating MEK/ERK pathway.

## DISCUSSION

It has been suggested that the augmented activation of c-kit/SCF signaling through a paracrine or autocrine mechanism in colorectal cancer closely correlate with its worse prognosis [12]. Therefore, we wondered whether the aberrant c-kit signaling was also involved in proliferation and invasion of the higher malignant CRMAC. In our murine model, the colorectal adenomas appeared at week 8 and the colorectal adenocarcinoma appeared at week 20, which resemble the adenoma-adenocarcinoma transformation in human colorectal cancer development [25]. It is worth noting that above half of WT mice developed CRMAC at 37 weeks, which showed higher grade malignant. The mucus was filled with MUC2, similar to human CRMAC [3, 26], suggesting that our CRMAC murine model was comparable with clinical CRMAC and feasible in this experiment. However, it should be pointed out that the occurrence rate of CRMAC (around 50%) in our study was somewhat inconsistent with data from clinic (15–20%). We considered that this was likely due to different tumorigenic processes in AOM+DSS induced murine model and spontaneous human colorectal cancer. In clinic, the tumorigenesis and progress are much more complicated involving inflammatory response, tumor microenvironment change etc [27, 28]. While, tumorigenic factor is relatively simple in AOM+DSS induced murine model, such as *apc* gene or *β-catenin* gene mutation [25]. This could be a possible explanation for the high occurrence rate of CRMAC in our study.

CRMAC in WT mice highly expressed c-kit, SCF and AKT than non-CRMAC in WT mice or Wads^−/−^ mice, suggesting that the aberrant activation of c-kit and its downstream AKT signaling might play a crucial role in the tumorigenesis of CRMAC. It has been reported that MUC2, which is transcriptionally directed by Math1 in mouse, a homologue of Hath1 in human, is often up-regulated in CRMAC [29, 30]. Given that we found distinct higher expressions of c-kit and AKT, we presumed that c-kit-activated AKT could inhibit Math1 degradation by inactivating GSK3β and subsequently promote MUC2 secretion as well as CRMAC tumorigenesis [31, 32]. With regard to how the c-kit/SCF signaling was highly activated, we did not think that gain-of-function mutation in *c-kit* or *scf* gene would be a contributor since we did not find *c-kit* or *scf* gene mutation in the AOM+DSS induced CRMAC (data not shown). It has been acknowledged that the aberrant c-kit/SCF paracrine or autocrine loop would lead to hyperactivation of c-kit signaling in small cell lung cancer, breast cancer and colorectal cancer [33]. In line with previous studies, we indeed found highly expressions of c-kit and SCF. Therefore, we took into account that the aberrant c-kit/SCF autocrine/paracrine stimulation loop could also promote the development of CRMAC.

It is well known that c-kit facilitates tumor growth, possibly through activating AKT pathway [10, 13, 24]. Consistently, our results showed that CRMAC in WT mice which exhibited the increased c-kit and AKT activities had larger size than non-CRMAC in Wads^−/−^ mice did. The activated AKT thereby decreased p53 and increased cyclin D1, which was well consistent with previous studies demonstrating less frequent p53 in CRMAC than in non-CRMAC [34, 35]. *In vitro* overexpression of c-kit in HCT-116 cells significantly accelerated cell proliferation accompanied with activated AKT, while blockage of c-kit activation showed opposite effects. These data provided a further evidence for ability of c-kit to enhance proliferation of CRMAC via activating AKT pathway.

Besides the key role of c-kit in proliferation of CRMAC, we investigated the potential effects of c-kit signaling on invasion of CRMAC. Interestingly, our *in vivo* and *in vitro* results demonstrated that c-kit positively regulated ETV4 expression. It is well accepted that ETV4 could promote transcriptions of its targets including MMPs and EMT associated genes in several kinds of tumors [20–22]. As expected, highly expressed ETV4 in CRMAC increased MMP-7, N-cadherin and vimentin whereas decreased E-cadherin, which promoted tumor invasion. Furthermore, our data suggested that phosphorylated ERK molecule enhanced ETV4 expression possibly through inhibiting ETV4 protein degradation instead of promoting ETV4 transcription, which needs future investigation. Taken together, it was reasonable to consider that c-kit-ERK-ETV4 axis might stimulate the metastasis and worse prognosis in CRMAC. A similar regulatory mechanism has been reported in GIST, in which c-kit promoted tumor progress through ERK-ETV1 axis [11].

**Figure 7:**
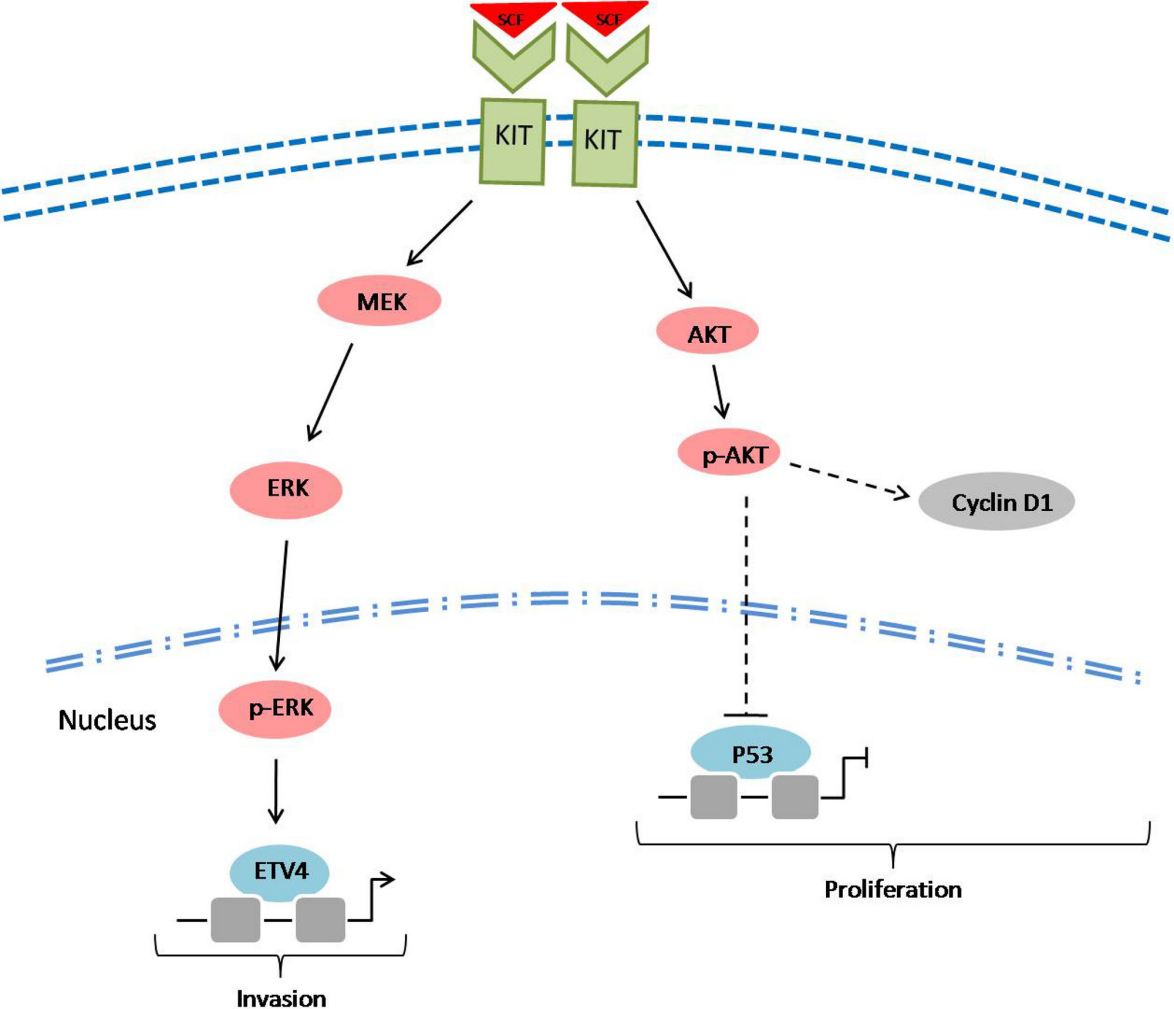
A scheme showing the potential role of c-kit signaling in the development of CRMAC SCF triggered c-kit signaling might increase cyclin D1 and decrease p53 through activating AKT pathway, thus enhance tumor cell proliferation. Furthermore, stimulated c-kit could increase ETV4 through activating MEK/ERK pathway, consequently confer a stronger capacity of tumor invasion.

In summary, the present study provided new insights into the role of c-kit signaling in the development of CRMAC (Figure 7). The highly activated c-kit signaling could stimulate AKT and ERK pathways, then facilitate the development of CRMAC which grew faster and more likely invaded into adjacent tissues. Although patients with CRMAC routinely receive the same first-line anti-tumor drugs as whom suffer from common colorectal cancer, it is still annoying for the poor chemotherapy sensitivity in CRMAC patients [35]. Therefore, targeting the c-kit signaling as well as its downstream molecules including AKT, ERK and/or ETVs might provide potential therapeutic benefits in the treatment of patients with CRMAC in clinic.

## MATERIALS AND METHODS

### Ethics statement

All animals' studies were carried out strictly under protocols approved by the Animal Care and Use Committee of Capital Medical University (Permit Number 2009-X-735). Every effort was made to minimize the number of animals used as well as their suffering.

### Animal and experiment design

Wads^−/+^ mice, which have a unique T-to-C transition mutation in *c-kit* gene that results in Phe-to-Ser substitution at amino acid 856 within a highly conserved tyrosine kinase domain [36], were purchased from the Model Animal Research Center of Nanjing University (Nanjing, China) and maintained in a specified pathogen-free environment with controlled conditions of humidity (40 ± 10%), 12/12 hr light/dark cycle and temperature (23 ± 2°C). Wild type (WT) and Wads^−/−^ mice were obtained by mating Wads^−/+^ parents as previously described [36]. Mice were genotyped by their distinct differences in fur pigmentation: black for WT, piebaldism for Wads^−/+^ and white for Wads^−/−^ mice. Adult WT (*n* = 45) and Wads^−/−^ (*n* = 20) mice (8–10 weeks old, 19–22 g) were intraperitonealy injected with azoxymethane (AOM, 10 mg/kg) (Sigma, USA) once and followed by three periods of 2.5% dextran sodium suffate (DSS) (MP Biomedicals, USA) in drink water (each period included administration of 2.5% DSS for 7 days, then given distilled water for 14 days). For the controls, WT mice (*n* = 5) received an injection of normal saline and had free access to distilled water. Mice were weighed twice weekly during the experiments and sacrificed at the indicated time points (4 weeks, 8 weeks, 20 weeks and 37 weeks) after AOM injection. After collection of whole colorectal tissues, the numbers and sizes of neoplasms in the entire large intestine were examined using a dissecting microscope. Half of neoplasms were frozen in liquid nitrogen for protein and mRNA detections and the rest of samples were fixed immediately in 4% paraformaldehyde for paraffin embedding or embedded in optimal cutting temperature (OCT) compound and then quickly frozen in liquid nitrogen.

### Cell culture and reagents

Human colorectal cancer cell line (HCT-116) was purchased from American Tissue Culture Collection (ATCC, USA) and cultured in DMEM (Life technologies, USA) supplemented with 10% fetal bovine serum (Life technologies, USA) and 1% penicillin/streptomycin (P/S, Life technologies, USA). Cells were grown at 37°C in the presence of 5% CO_2_. The medium was replaced every 48 hrs. Cells used in the experiments were between passages 5 and 10. Imatinib, recombinant human SCF (rhSCF), U0126 and MG132 were obtained from Biovision (USA), R&D Systems (USA), Cell Signaling Technology (USA) and Sigma (USA), respectively.

### Histopathological examination

Sections (5 μm) were cut from the paraffin embedded tumor tissues, then stained with either HE (hematoxylin and eosin) for histological observation or Alcian blue for mucus area assessment. Tumor malignancy was histopathologically defined based on the marked architectural changes, including back-to-back gland configuration, amount of nuclear division and marked cytological atypia.

### Immunofluorescent staining

Frozen sections (7 μm) were cut from tumor tissues embedded in OCT using a cryostat (Leica CM3050S, Germany). For cultured cells, HCT-116 cells were grown on coverslips in 24-well plates. Sections or cultured cells were fixed with 100% acetone for 10 min or 4% paraformaldehyde for 20 min at 25°C. Non-specific binding sites were blocked with 1% bovine serum albumin (BSA, Sigma, USA) for 30 min. Then specimens were incubated with the corresponding antibodies (Supplementary Table S2) at 4°C overnight, washed three times with PBS (5 min each), then incubated with Cy3-conjugated corresponding secondary antibody for 1 hr at 25°C in the dark. Specimens were stained with DAPI (Zhongshanjinqiao Biotechnology, China) for identifying nuclei, and observed with a fluorescence microscope (Nikon, Eclipse Ni, Japan). Specimens incubated with an isotype control antibody and omission of primary antibody were used as negative controls, respectively.

### Lentiviral vector construction and infection

To construct lentivirus vector mediated *c-kit* (Lv-*c-kit*), the CDS of *c-kit* gen*e* was chemically synthesized by the technical support from GeneChem and inserted into a lentiviral vector (GV287, GeneChem, China, Supplementary Figure S2). HCT-116 cells were seeded in 6-well plate at 5 × 10^4^/well and infected with Lv-*c-kit* when 30% confluency. Three days post-infection, the efficiency of infection was evaluated by observing the EGFP expression with an inverted fluorescence phase contrast microscope (Leica DMI3000 B, Germany).

### Real-time monitoring the cell proliferation

The “xCELLigence” system (ACEA Biosciences, USA), consisting of E-plates and Real Time Cell Analyzer Dual Purpose (RTCA-DP) instrument was employed to monitor cell proliferation by measuring cell index (CI) which are proportional to the number of cells [37]. Briefly, cells were seeded in E-plates at a density of 8000 cells/well. After that, the E-plates were transferred to the RTCA-DP instrument for automated real-time monitoring at standard incubator conditions, with readouts of the CI every 15 min. Each experiment was independently performed 4 times.

### Real-time monitoring the cellular invasion

Cell invasion were also monitored using the “xCELLigence” system, but performed in Cell Invasion-and-Migration (CIM)-plates rather than in E-plates [37]. Briefly, cells were seeded in the upper chambers (20,000 cells/well) of CIM-plates, which were loaded with 30 μL of 10% Matrigel (BD Biosciences, USA) to create a 3D biomatrix film and were filled with serum-free medium. Bottom chambers were filled with serum-containing medium to promote invasion across membranes towards the serum gradient. After seeding, the CIM-plates were transferred into the RTCA-DP instrument for real time read-outs. Impedance or CI was registered only from cells capable of invading through the membranes. Four independent experiments were performed.

### RNA extraction and real-time PCR

Total RNA was extracted from cultured cells with TRIzol reagent (Sigma, USA), according to the manufacturers' instructions. RNA concentration was measured using Nanodrop 2000c spectrophotometer (Thermo Scientific, USA). Reverse transcription reactions were performed using Super cDNA First-Strand Synthesis Kit (CWBiotech, China). The 15 μl reactions were incubated in a Veriti 96 well Thermal Cycler (Applied Biosystem, USA) for 40 min at 42°C and 5 min at 85°C. Real-time PCR was performed in an ABI 7500 real-time PCR system (Applied Biosystem, USA) using Ultra SYBR Mixture with ROX (CWBiotech, China). The following primers were used: *ETV4* (Forward: GCCCATTTCATTGCCTGGAC; Reverse: GACTTGCCATTTCTCCACTTTCC); *GAPDH* (Forward: AGAAGGCTGGGGCTCATTTG, Reverse: AGGGGCCATCCACAGTCTTC). The 25 μl reactions were incubated at 95°C for 10 min, followed by 40 cycles at 95°C for 15 s and at 60°C for 1 min. All reverse transcription reactions included no-template controls; and all PCR reactions were run in triplicate. Relative gene expression was determined using comparative C_T_ (2^−ΔΔCt^) method.

### Western blot analysis

Total proteins were extracted from cultured cells or tumor samples using the RIPA lysis buffer containing protease inhibitors (Applygen, China) and phosphatase inhibitors (Sigma, USA). The protein concentrations were determined by NanoDrop 2000c spectrophotometer using BCA protein assay kit (Applygen, China). After loading equal amount of protein samples, SDS-PAGE (12% sodium dodecyl sulfate polyacrylamide gel electrophoresis) was performed. The proteins were then transferred to a PVDF membrane (Millipore, USA). After blocking with Tris-buffered saline containing 0.05% Tween-20 (TBST) and 5% non-fat dry milk or 5% BSA for 1 hr, the membrane was incubated with the corresponding antibodies (Supplementary Table S2) at 4°C overnight, washed in TBST, followed by incubation with the corresponding horseradish peroxidase-conjugated secondary antibodies (Santa Cruz, USA) for 1 hr. Visualization of the proteins was detected with ECL chemiluminescence. Beta-actin (Santa Cruz, USA) was used as a loading control. The intensity values were assessed and analyzed with Image J software (NIH).

### Statistical analysis

The software package SPSS 13.0 (Chicago) was used for all data analysis. In general, results among experimental groups were analyzed by student's *t*-test or one-way ANOVA. Data were presented as the means ± SEM. For all tests, *p*-value <0.05 was considered statistically significant.

## SUPPLEMENTARY FIGURES AND TABLES


