# Supplementary material for: C-kit signaling promotes proliferation and invasion of colorectal mucinous adenocarcinoma in a murine model
Source: Oncotarget. 2015 Sep 2;6(29):27037–48. doi: 10.18632/oncotarget.4815 (PMC4694972; doi:10.18632/oncotarget.4815)
Supplement: Supplementary file 1 [file oncotarget-06-27037-s001.pdf]

SUPPLEMENTARY FIGURES AND TABLES

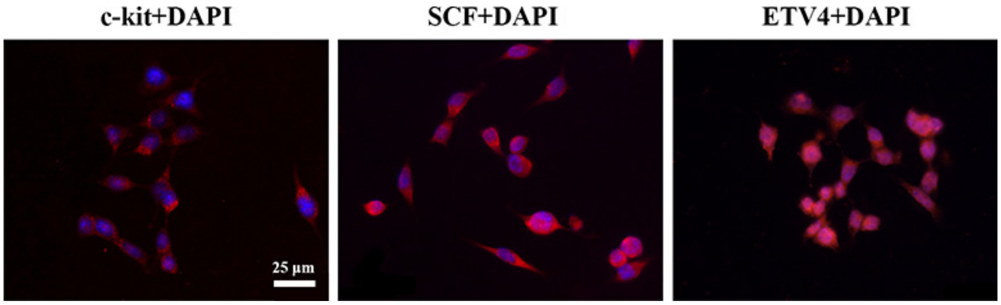

Supplementary Figure S1: Immunofluorescence showing that HCT-116 cells express c-kit, SCF and ETV4.

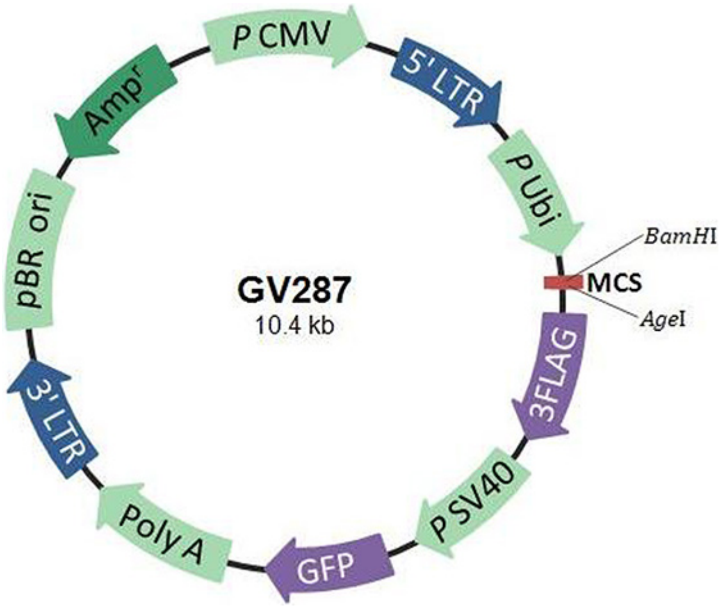

Supplementary Figure S2: Vector GV287 used in the present study.

Supplementary Table S1: 37 weeks after AOM injection, the mucus areas within tumors collected from WT mice and Wads mice, respectively

Time	Group	Average mucus area (dpi)	Average tumor area (dpi)	Mucus area/ Tumor area
37 weeks	WT (CRMAC)	3974032	5510185	72.12%
	WT (CRMAC)	1496329	2464031	60%
	WT (CRMAC)	1967032	3055598	64%
	WT (CRMAC)	489445	7968579	61%
	WT (CRMAC)	3019783	5376586	56%
	WT (CRMAC)	3126768	5645814	55%
	WT (CRMAC)	1575856	3094082	51%
	WT (CRMAC)	3038242	6038447	50%
	WT (non-CRMAC)	304007	3248282	9.36%
	WT (non-CRMAC)	68698	3870512	1.17%
	WT (non-CRMAC)	90096	2698903	3.34%
	WT (non-CRMAC)	102857	972742	10.91%
	WT (non-CRMAC)	195756	5511867	21.3%
	WT (non-CRMAC)	3186826	9263700	35%
	WT (non-CRMAC)	435872	3885219	12%
	$Wads^{-/-}$ (non-CRMAC)	732619	4823561	15.19%
	$Wads^{-/-}$ (non-CRMAC)	27821	2758826	1.01%
	$Wads^{-/-}$ (non-CRMAC)	94930	2805232	3.38%
	$Wads^{-/-}$ (non-CRMAC)	40856	2668515	1.53%
	$Wads^{-/-}$ (non-CRMAC)	229819	698619	33%

Supplementary Table S2: Antibodies

	Immunohistochemical staining	Western blot analysis
rabbit anti-ETV4 (Santa Cruz, USA)	1/500	1/1000
rabbit anti-MUC2 (Santa Cruz, USA)	1/200	
rabbit anti-E-cadherin (Cell Signaling Technology, USA)		1/2000
mouse anti-SCF (Santa Cruz, USA)	1/200	1/1000
rat anti-c-kit (eBioscience, USA)	1/400	
rabbit anti-c-kit (Cell Signaling Technology, USA)		1/1000
rabbit anti-phospho-c-kit (Cell Signaling Technology, USA)		1/500
rabbit anti-Erk1/2 (Cell Signaling Technology, USA)		1/2000
rabbit anti-phospho-Erk1/2 (Cell Signaling Technology, USA)		1/5000
goat anti-MMP-7 (Santa Cruz, USA)		1/1000
rabbit anti-AKT (Cell Signaling Technology, USA)		1/1000
rabbit anti-phospho-AKT (Cell Signaling Technology, USA)		1/2000
rabbit anti-N-cadherin (Santa Cruz, USA)		1/1000
rabbit anti-Vimentin (Santa Cruz, USA)		1/1000
mouse anti-P53 (Cell Signaling Technology, USA)		1/1000
rabbit anti-Cyclin D1 (Cell Signaling Technology, USA)		1/1000
mouse anti-beta-actin (Santa Cruz, USA)		1/6000
